# Measurement error and bias in real-world oncology endpoints when constructing external control arms

**DOI:** 10.3389/fdsfr.2024.1423493

**Published:** 2024-07-19

**Authors:** Benjamin Ackerman, Ryan W. Gan, Craig S. Meyer, Jocelyn R. Wang, Youyi Zhang, Jennifer Hayden, Grace Mahoney, Jennifer L. Lund, Janick Weberpals, Sebastian Schneeweiss, James Roose, Juned Siddique, Omar Nadeem, Smith Giri, Til Stürmer, Sikander Ailawadhi, Ashita S. Batavia, Khaled Sarsour

**Affiliations:** ^1^ Janssen Research and Development, LLC, A Johnson and Johnson Company, Raritan, NJ, United States; ^2^ Department of Epidemiology, University of North Carolina, Chapel Hill, United States; ^3^ Division of Pharmacoepidemiology and Pharmacoeconomics, Brigham and Women’s Hospital, Harvard Medical School, Boston, United States; ^4^ Flatiron Health, New York, NY, United States; ^5^ Preventive Medicine and Psychiatry and Behavioral Science, Feinberg School of Medicine, Northwestern University, Chicago, United States; ^6^ Department of Hematology and Oncology, Dana-Farber Cancer Institute, Harvard Medical School, Boston, United States; ^7^ Division of Hematology and Oncology, Department of Medicine, University of Alabama at Birmingham, Birmingham, United States; ^8^ Department of Hematology, Mayo Clinic, Jacksonville, United States

**Keywords:** measurement error, real-world data (RWD), oncology, external control arm, misclassification bias, surveillance bias, progression-free survival, endpoints

## Abstract

**Introduction:** While randomized controlled trials remain the reference standard for evaluating treatment efficacy, there is an increased interest in the use of external control arms (ECA), namely in oncology, using real-world data (RWD). Challenges related to measurement of real-world oncology endpoints, like progression-free survival (PFS), are one factor limiting the use and acceptance of ECAs as comparators to trial populations. Differences in how and when disease assessments occur in the real-world may introduce measurement error and limit the comparability of real-world PFS (rwPFS) to trial progression-free survival. While measurement error is a known challenge when conducting an externally-controlled trial with real-world data, there is limited literature describing key contributing factors, particularly in the context of multiple myeloma (MM).

**Methods:** We distinguish between biases attributed to how endpoints are derived or ascertained (misclassification bias) and when outcomes are observed or assessed (surveillance bias). We further describe how misclassification of progression events (i.e., false positives, false negatives) and irregular assessment frequencies in multiple myeloma RWD can contribute to these biases, respectively. We conduct a simulation study to illustrate how these biases may behave, both individually and together.

**Results:** We observe in simulation that certain types of measurement error may have more substantial impacts on comparability between mismeasured median PFS (mPFS) and true mPFS than others. For instance, when the observed progression events are misclassified as either false positives or false negatives, mismeasured mPFS may be biased towards earlier (mPFS bias = −6.4 months) or later times (mPFS bias = 13 months), respectively. However, when events are correctly classified but assessment frequencies are irregular, mismeasured mPFS is more similar to the true mPFS (mPFS bias = 0.67 months).

**Discussion:** When misclassified progression events and irregular assessment times occur simultaneously, they may generate bias that is greater than the sum of their parts. Improved understanding of endpoint measurement error and how resulting biases manifest in RWD is important to the robust construction of ECAs in oncology and beyond. Simulations that quantify the impact of measurement error can help when planning for ECA studies and can contextualize results in the presence of endpoint measurement differences.

## 1 Introduction

Randomized controlled trials (RCTs) remain the gold standard for the investigation of treatment efficacy (Meinert, 1996; Sibbald and Roland, 1998; Meldrum, 2000; Hariton and Locascio, 2018). However, in situations where a randomized design is not feasible due to ethical reasons, challenges in enrollment of trial participants (e.g., in cases of rare disease, or other highly specialized populations) or lack of clinical equipoise, there is an increasing interest in the construction of external control arms (ECAs) for comparison to a single-arm trial (Schmidli et al., 2020; Carrigan et al., 2022; Oksen et al., 2022; U.S. Department of Health and Human Services, Food and Drug Administration, 2023). An ECA may be identified from real-world data (RWD) sources collected outside of the trial such as electronic health records, registries, or administrative claims data. Challenges from potential biases, namely, bias due to measurement error (i.e., misclassification bias, surveillance bias), between RWD and RCTs have limited the use and acceptance of ECAs as comparators to trial populations (Center for Biologics Evaluation and Research, U.S. Food and Drug Administration, 2021; Webster-Clark et al., 2020). Given these biases, careful consideration must be given to align populations and clinical endpoints in the trial and real-world (Seeger et al., 2020; LoCasale et al., 2021).

Oncology clinical trials commonly use a primary endpoint of progression-free survival (PFS), the earliest time from the start of treatment to a progression event or death, which is a widely accepted surrogate for overall survival (OS) (U.S. Department of Health and Human Services, Food and Drug Administration, 2018; Lin et al., 2023). Progressive disease can be measured and determined in different ways for different cancer types; for example, imaging is a common and accepted modality for assessing progressive disease in solid tumor types (Eisenhauer et al., 2009). In multiple myeloma (MM), a cancer that forms in plasma cells, disease progression is determined in accordance with the International Myeloma Working Group (IMWG) Treatment Response criteria, which is based on results of blood, urine, and bone marrow assays, as well as imaging techniques that evaluate bone lesion (Kumar et al., 2016). These key biomarkers for disease assessment are typically collected routinely in a trial setting per a protocol-defined schedule; however, in real-world settings, the collection and timing of these biomarkers and imaging assessments may vary, and their availability in RWD may be affected by data capture and normalization processes. These differences in how and when disease assessments occur in the real-world may lead to measurement error, and may limit the comparability of real-world PFS (rwPFS) to trial PFS by introducing misclassification bias and surveillance bias, respectively.

While measurement error is a known challenge when augmenting single-arm trials with RWD, there is limited literature describing factors that contribute to measurement differences (e.g., misclassified events, irregular assessment frequencies), particularly in the context of MM. Furthermore, much is still unknown regarding the potential impact of these types of measurement error in different contexts and how they interplay. The aim of this study is to investigate key sources of measurement error that contribute to bias when estimating rwPFS and illustrate how they may impact the comparability with trial PFS using a simulation example. The sections of this paper are as follows: First, we define the measurement error types of interest and provide framing for how their related biases may manifest in RWD. We then conduct a simple simulation study to illustrate how these biases may behave, both independently as well as together. We conclude by highlighting considerations on how measurement error may impact the estimation of rwPFS and discuss the importance in further quantifying bias due to these errors in practice when comparing real-world and clinical trial endpoints.

## 2 Methods and materials

### 2.1 Defining types of bias due to endpoint measurement error

In this section, we disaggregate bias of rwPFS endpoints due to measurement error into misclassification bias and surveillance bias. The former describes a bias attributed to *how* the endpoint is *derived* or *ascertained*, such that the true disease status may not be observed. The latter describes a bias attributed to *when* outcomes are *observed* or *assessed*, namely, at a different (and irregular) interval than a trial. In the context of ECAs, it is important to note that these biases are defined in *relation to the trial* population as the source of “truth.” In other words, here, we refer to biases that are attributed to differences between the RWD and the trial approaches to disease evaluation, which present when using RWD *in lieu* of a randomized trial’s control arm as a comparator. We now define these biases and discuss attributes of RWD that contribute to them and highlight how they manifest in the context of MM.

#### 2.1.1 Misclassification bias

At each disease assessment time point, a patient’s progressive disease status can be misclassified in one of two ways: *false negatives* are when patients experience progression events, but the events are not captured or observed, whereas *false positives* are when patients do not experience progression events but are falsely classified as having progressed at a certain time. These misclassification errors can impact the observed time to first progression, which in turn affects how the PFS endpoint is constructed. More specifically, false negative events may lead to longer observed PFS times, while false positive events may lead to shorter observed PFS times. It is important to note that false negatives are only possible among patients who truly progress, and thus, the impact of such errors on bias in the PFS endpoint are also dependent on the disease setting and true progression event rate. For example, in disease settings where true progression event rates within a typical duration of follow-up are low (e.g., newly diagnosed multiple myeloma, or NDMM), fewer false negative errors are possible, and therefore false positive errors are more likely to drive overall bias when estimating median PFS.

Similarly, misclassification of progression events may not always introduce bias in the PFS endpoint, and may depend on the amount of time bias attributed to the error types (Bakoyannis and Yiannoutsos, 2015; Edwards et al., 2023). For example, if a progression event is not captured (i.e., there is a false negative), but the patient has a death event that occurs a few weeks later, then the observed “mismeasured” PFS may only be biased by a few weeks. Alternatively, if a progression event is falsely detected (i.e., there is a false positive) many months before a patient truly progresses, then their PFS time may be more substantially biased.

In MM RWD, there may be high rates of missingness among biomarkers required to derive progression according to full IMWG criteria due to real-world care patterns (e.g., the urine protein electrophoresis test, UPEP, requires a patient to collect urine over 24-h), which limits real-world data use (Foster et al., 2018). Furthermore, data missingness in RWD may reflect an absence of test collection, or it may reflect tests that are collected but not observed or captured in the data source (Sondhi et al., 2023; Vader et al., 2023), and thus the full IMWG criteria for deriving progression may not be feasible to implement. Flexible alternate algorithms for deriving endpoints may be used instead; these alternative algorithms are based on IMWG criteria but are designed to be more accommodating of real-world lab collection practices or missingness rates (Foster et al., 2019). Application of these alternative algorithms may lead to misclassification of progression events relative to the full IMWG criteria as they would be applied in a clinical trial setting. While it may be possible to make minor improvements or alterations to how real-world progression is derived in MM, an “error-free” flexible algorithm may not be achievable considering differences in the underlying data availability and completeness as well as clinical practice.​

#### 2.1.2 Surveillance bias

In a clinical trial setting, patients are assessed according to a protocol-specified schedule (e.g., on a bi-monthly or monthly frequency). While it is possible for patients to have a progression event or clinical worsening in between scheduled assessments, progression events are typically detected when a patient returns for their subsequent visit. This may lead to a delay between when a progression event truly occurred and when it was observed, otherwise referred to as surveillance bias (Panageas et al., 2007). Such delays in event detection can depend on the length of the assessment intervals (i.e., if patients are assessed more frequently, the time between event occurrence and event detection may be shorter) (Kapetanakis et al., 2019; Adamson et al., 2022; Zhu and Tang, 2022).

In a randomized controlled trial, patients in both arms follow the same assessment schedule, and therefore any event detection delays may be assumed to be similar across arms. Therefore, such event detection delays may not impact treatment effect estimates. However, in the context of ECAs, patients in the external comparator may be assessed on a *different* frequency than the internal arm. Such differences in assessment schedules may lead to biased estimates when comparing the two arms.

In contrast to trials, patients in the real-world setting are not always assessed according to a strict schedule. It is possible that, on average, patient visits are distributed with some degree of consistency, albeit likely with much higher variability than in a trial. Irregular assessment frequencies may therefore be observed in RWD, and this could be for several reasons: 1) RWD patient populations are often quite heterogeneous, so there may be variations in how often patients come in for visits based on site or clinical practice, geographic proximity, or socio-economic factors, and 2) patient visits may be driven by symptoms, management of co-morbid conditions, convenience or other factors, and clinicians may recommend that patients schedule their subsequent appointments sooner or later accordingly. When conducting an externally-controlled trial with RWD, such differences in assessment timing may contribute to biased endpoint comparisons.

## 3 Simulation study

We now describe a simulation study to illustrate the association between misclassification of progression events, irregular assessment frequencies, and biases due to these errors in the PFS endpoint. Let N denote the total number of patients in our external comparator. For all N patients, we start by simulating true times to death (for OS), PFS and end of follow-up (FUP) using independent exponential distributions where the rates are defined by the desired median times of mOS, mPFS, and mFUP, respectively:

$$\begin{array}{rrr} T_{OS}∼\mathrm{Exp}\left(\lambda_{OS}\right), & & \lambda_{OS}=\log\left(2\right)/\text{mOS}\\ T_{PFS}∼\mathrm{Exp}\left(\lambda_{PFS}\right), & & \lambda_{PFS}=\log\left(2\right)/\text{mPFS}\\ T_{FUP}∼\mathrm{Exp}\left(\lambda_{FUP}\right), & & \lambda_{FUP}=\log\left(2\right)/m\text{FUP} \end{array}$$

To simulate patients’ true time from treatment initiation to first progression, $T_{prog}$ , we compare their simulated PFS time with their simulated OS time and derive it as follows:

$$T_{prog}=\left\{\begin{array}{lll} T_{PFS} & \text{if} & T_{PFS}<T_{OS}\\ NA & \text{if} & \text{otherwise} \end{array}\right.$$

Since PFS is a composite of time to death and time to first progression, this allows us to determine if the simulated PFS time is attributed to a progression event or a death event; if a patient’s PFS time is earlier than their OS time, then we can infer that a progression event occurred. Otherwise, we can infer that the PFS time is attributed to a death event.

Once underlying true progression event times are simulated, the events are mapped to a fixed trial-like disease assessment schedule, such that progression events are only observed when patients are simulated to be evaluated by clinicians per protocol:

$${\bar{T}}_{prog}=\lceil \frac{T_{prog}}{d}\rceil \times d$$

where *d* is the per-protocol time between assessments and ⌈ ⌉ is the “ceiling” function that rounds the contents up to the nearest whole number. For example, if a patient’s $T_{prog}$ is simulated to occur on day 53, and they are assessed every 28 days per trial protocol (e.g., d=28 ), then their true progression event will be observed on day $\lceil \frac{53}{28}\rceil \times 28=56$ , since $\lceil \frac{53}{28}\rceil =\lceil 1.89\rceil =2$ .

Patients’ true PFS times are then constructed as $\min\left({\bar{T}}_{prog},T_{OS},T_{FUP}\right)$ and event indicators are determined by this time. Next, we describe how we introduce misclassification of progression events and irregular assessment times via simulation.

### 3.1 Simulating misclassification errors and mismeasured PFS times

To simulate misclassification errors, we assume that a flexible alternative IMWG algorithm to derive progression events with known sensitivity and specificity has been applied. We define sensitivity and specificity based on 1) whether patients’ true PFS time is equal to their mismeasured PFS time (i.e., PFS constructed using progression real-world derived progression events) and 2) whether patients’ true PFS time is determined by a progression event (versus death or censoring) as follows:

Sensitivity = P (true PFS = mismeasured PFS | true PFS = time to first progression)

Specificity = P (true PFS = mismeasured PFS | true PFS = time to death or censoring)

Let $N_{\text{pfs}‐\text{prog}}$ denote the number of patients for whom PFS is defined by a progression event. Recall that only patients who truly have PFS defined by a progression event can be classified as a false negative. To simulate *false negatives*, we simulate $N_{\text{pfs}‐\text{prog}}$ Bernoulli events with probability $p_{fn}=\left(1-\text{sensitivity}\right)$ . To simulate *false positives*, we simulate $N-N_{\text{pfs}‐\text{prog}}$ Bernoulli events with probability $p_{fp}=\left(1-\text{specificity}\right)$ .

Next, we generate the mismeasured time to progression based on the misclassification type.

For *false negative patients*, we simulate the time as:

$$T_{prog}^{mis}=T_{prog}+T_{\text{fn}\;\text{bias}}$$

where $T_{\text{fn}\;\text{bias}}∼\mathrm{Exp}\left(\lambda_{PFS}\right)$ . In other words, for each false negative patient, we add random exponentially distributed time to their time to progression.

For *false positive patients*, we simulate the time as:

$$T_{prog}^{mis}=T_{PFS}-T_{\text{fp}\;\text{bias}}$$

where $T_{\text{fp}\;\text{bias}}∼U\left[0,T_{PFS}\right]$ . In other words, for each false positive patient, we generate a progression event that falsely happened *any time* between treatment initiation and their true PFS time.

When simulating the impact of misclassification bias only (i.e., no irregular assessment frequency), mismeasured time to progression is mapped to the trial protocol assessment schedule as $\bar{T_{prog}^{mis}}=\lceil \frac{T_{prog}^{mis}}{d}\rceil \times d$ . This is then used to construct the mismeasured PFS endpoint as $\min\left(\bar{T_{prog}^{mis}},T_{OS},T_{FUP}\right)$ and mismeasured event indicators are determined accordingly.

### 3.2 Simulating irregular assessment schedules and observed PFS times

As described above, we assume that disease assessments for progression in a trial follow a strict disease assessment schedule of every *d* days per protocol. In RWD, on the other hand, we assume that a patient is assessed on an *irregular* schedule, where the mode of time between visits is *d* days, but with greater variability than the trial. For example, RWD patients may be assessed *roughly* every 28 days, but may, on occasion, have visits that are more (or less) spread out (Foster et al., 2018; Roose et al., 2022). To simulate irregular times between assessments, assuming a trial-like mode with greater variability, we use a mixture of distributions that contains an identifiable mode, but with variability that may be characterized by another distribution. Here, we will use a log-normal mixture distribution:

$$g(x;\mu_{1},\sigma_{1},\mu_{2},\sigma_{2},p)=(1-p)f(x;\mu_{1},\sigma_{1})+\mathrm{pf}(x;\mu_{2},\sigma_{2})$$

Where *g* is the mixture function, *f* is the lognormal function, p = probability of assessment being off-cycle (i.e. deviating from the mode of *d* days), *μ*_1_ and *σ*_1_ represent the log mean day and standard deviation of the “on-assessment” day and *μ*_2_ and *σ*_2_ represent the log mean and standard deviation of “off-cycle” days.

For each simulated patient, we simulate a vector of assessment times according to this mixture distribution. Then, for patients who have been simulated to have a progression event, we shift their simulated true event time to equal the first irregular assessment occurring after the event. For example, if a patient's *T_prog_* = 140 days, and we simulate irregular assessment times for them at days 27, 145, 171, 184 and 217, then their mismeasured progression event time would be shifted to day 145.

### 3.3 Simulation scenarios

In order to illustrate the potential impacts of and interplay between these measurement error biases, we consider a data generating model using parameters defined in Table 1, based on the control arm of a historical trial conducted among patients with NDMM that received lenalidomide and dexamethasone (Facon et al., 2021). Table 2 describes the scenarios of interest, varying frequencies of false positive (1—specificity) and false negative (1—sensitivity) errors, as well as the type of assessment frequency that patients follow. Parameter values were selected based on prior feasibility analyses and clinical perspectives regarding the performance of flexible alternative IMWG algorithms, as well as the frequency of patient assessments, in RWD. By studying simulation results under perfect sensitivity and specificity (Scenario 1), we can quantify biases attributed to differences in assessment frequency. By studying simulation results under trial-like assessment frequencies (Scenarios 2–4), we can quantify biases attributed to misclassification errors alone. Simulating both together (Scenario 5) will demonstrate how these biases may manifest jointly. Note that when we simulate both biases, we begin by first introducing misclassification, followed by irregular frequency.

**TABLE 1:** Parameters used to simulate “true” NDMM population.

Parameter	Description	Parameter value
N	Sample size	365
mOS	Median overall survival time (months)	66.4
mPFS	Median progression-free survival time (months)	34.2
mFUP	Median follow-up time (months)	56.2
end_of_study	End of study period (months)	78.6

**TABLE 2:** Parameters used to define mismeasurement of progression events and assessment time.

Scenario	Sensitivity	Specificity	Assessment frequency
1	1.0	1.0	Irregular, RWD-like
2	0.5	1.0	Per trial protocol, every 28 days
3	1.0	0.8	Per trial protocol, every 28 days
4	0.5	0.8	Per trial protocol, every 28 days
5	0.5	0.8	Irregular, RWD-like

For each simulation iteration, we generate two samples–one with the outcome measured “correctly” and the other “mismeasured” with error. We define bias as the difference in median mismeasured (i.e., “real-world”) PFS and underlying true (i.e., “trial”) PFS, obtained via Kaplan-Meier (KM) estimation. Positive bias denotes mismeasured PFS is longer, on average, than true PFS, while negative bias denotes mismeasured PFS is shorter than true PFS. We run 1,000 iterations of each simulation scenario and report confidence intervals as the 2.5th and 97.5th quantiles of the bias distributions. We also estimate the “False Discovery Rate” as the proportion of simulation iterations for which the true and mismeasured PFS KM curves are statistically different (defined by a *p*-value <0.05 via the log-rank test).

## 4 Results

Simulation results are presented in Table 3, and the key findings are summarized below.

**TABLE 3:** Simulation results varying misclassification rates and assessment frequencies.

Scenario	Sensitivity	Specificity	Assessment frequency	mPFS bias (95% CI), in months	% bias (mPFS bias/true mPFS) (95% CI)	False discovery rate (%)
1	1.0	1.0	Irregular, RWD-like	0.67 (−7.4, 8.7)	2.0% (−21.6%, 25.4%)	5.3
2	0.5	1.0	Per trial protocol, every 28 days	13 (3.7, 22)	38% (10.8%, 64.3%)	88.2
3	1.0	0.8	Per trial protocol, every 28 days	−6.4 (−14, 0.93)	−18.7% (−41%, 2.7%)	56.8
4	0.5	0.8	Per trial protocol, every 28 days	4.8 (−3.7, 13)	14% (−10.8%, 38%)	15.9
5	0.5	0.8	Irregular, RWD-like	5.9 (−2.3, 15)	17.3% (−6.7%, 43.9%)	27.6

### 4.1 Surveillance bias only (no misclassification)

**Simulation scenario 1:** First, let’s consider the scenario where progression events are detected without any error (sensitivity and specificity = 1), but they are assessed by a clinician on an irregular frequency that is more variable than a trial protocol. Introducing surveillance bias in the form of differing assessment frequencies biases the observed mPFS towards longer times (mPFS bias = 0.67 months, 95% CI: −7.4 to 8.7 months), albeit this does not translate to statistical differences between the true and mismeasured PFS curves (False Discovery Rate = 5.3%).

### 4.2 Misclassification bias only (no surveillance bias)

Next, let’s consider scenarios where patients are assessed per a trial assessment schedule, but patients’ progression events are misclassified as either false positives or false negatives.

**Simulation scenario 2:** When sensitivity is 50% and specificity is 100%, mismeasured mPFS is substantially biased towards longer times than the true PFS (mPFS bias = 13 months, 95% CI: 3.7 to 22 months), and there is a statistical difference between the true and mismeasured PFS curves almost 90% of the time. In other words, if a flexible alternate algorithm to derive progression in the real-world misses 50% of patients who truly progress, but does not introduce any false progression events, then such an algorithm can yield real-world mPFS that appears much longer than the truth.

**Simulation scenario 3:** When sensitivity is 100% and specificity is 80%, mismeasured mPFS is biased towards *shorter* times than the true PFS (mPFS bias = −6.4 months, 95% CI: −14 to 0.93 months), and there is a statistical difference between the true and mismeasured PFS curves ∼57% of the time. This represents a scenario where a real-world approach to derive progression captures all true progressors but overclassifies progression for those who do not truly progress.

**FIGURE 1:**
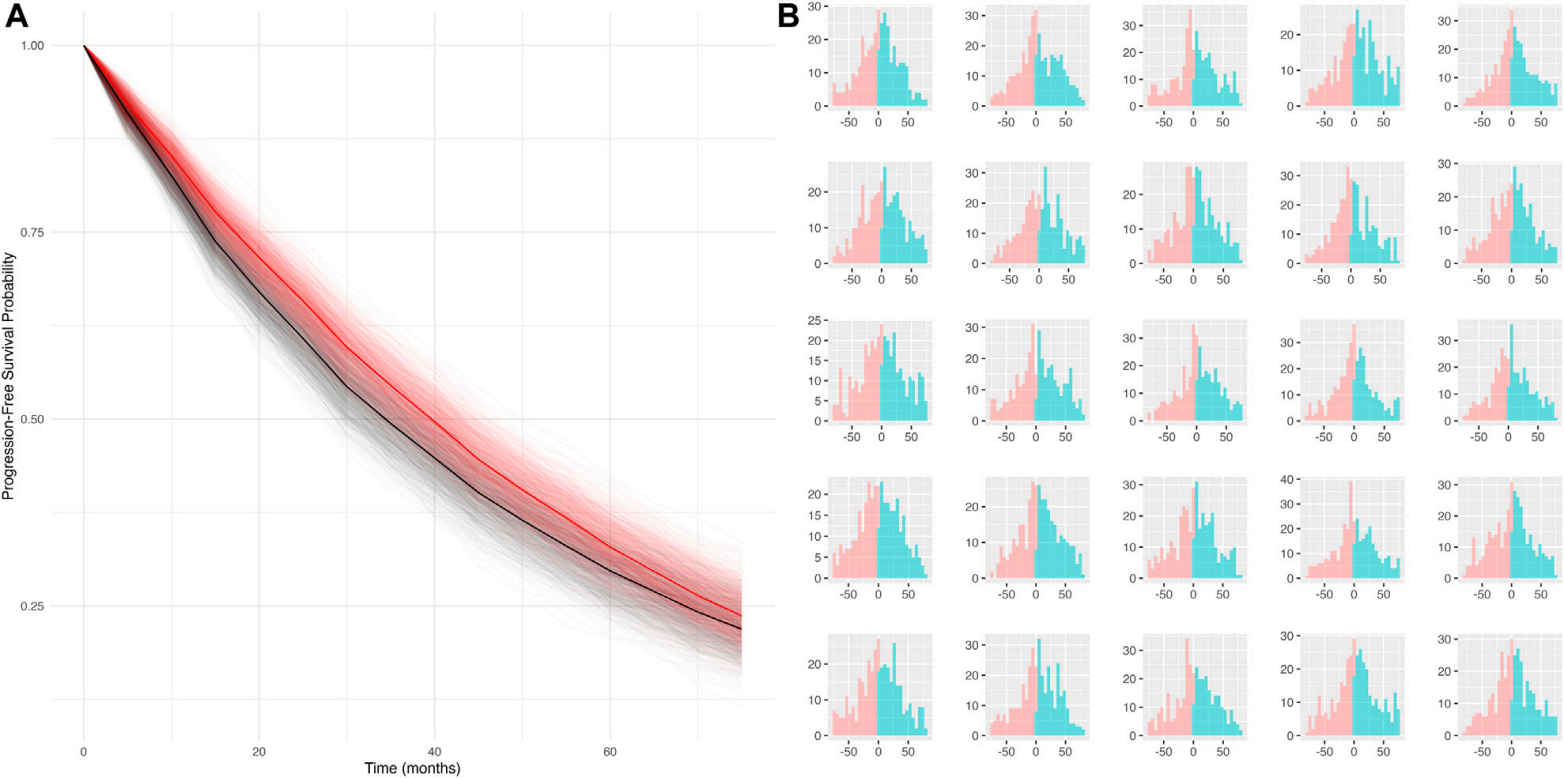
**(A)** Simulated KM curves comparing mismeasured (red) and true (black) PFS curves. Bold lines represent the average across all simulation runs. **(B)** Person-bias in months (true PFS–mismeasured PFS) for 25 randomly sampled simulated datasets. Bars in blue denote bias attributed to false negatives, bars in red denote bias attributed to false positives.

**Simulation scenario 4:** When both false positives *and* false negatives are simulated together (sensitivity = 50%, specificity = 80%), the bias is smaller (mPFS bias = 4.8 months, 95% CI: −3.7 to 13 months), and the false discovery rate is reduced (statistical differences between true and mismeasured PFS curves detected ∼16% of the time). Furthermore, the PFS Kaplan-Meier curves for the true and mismeasured outcomes appear to overlap in this scenario, suggesting that the two endpoint versions may be more comparable (See Figure 1A). However, upon further inspection, these errors are each yielding their own substantial biases that appear to oppose one another (See Figure 1B).

### 4.3 Misclassification and surveillance bias

**Simulation scenario 5:** Lastly, let’s consider the scenario where misclassification and surveillance biases are both present. Introducing irregular assessment frequency, on top of false positives and negatives of progression events, further biases mPFS to later times (mPFS bias = 5.9 months, 95% CI: −2.3 to 15 months), and the probability of detecting a statistical difference between true and mismeasured PFS curves increases from 15.9% to 27.6%. While irregular frequency alone had limited impact on the bias, the results in this scenario highlight that the joint contribution of misclassification and surveillance biases (the most realistic real-world scenario) may be more substantial.

## 5 Discussion

When developing an ECA to contextualize the findings of a single-arm trial, it is important to consider how measurement differences can affect endpoint comparability and the potential for bias. In this research, we have defined sources of measurement error that may add bias and limit comparability of real-world and trial endpoints in MM and we have highlighted several key contributors to measurement differences in PFS. Through simulation, we have illustrated how differences between real-world mPFS and trial mPFS may be attributed to misclassification bias as well as surveillance bias in this disease population.

Even when measurement error is present among individual patients, simulations suggest that such bias may not always result in observable or statistical differences between true (i.e., trial) and mismeasured (i.e., real-world) mPFS estimates. For example, simulations showed that when progression events are classified correctly, but patients have irregular assessment times (Scenario 1), a small amount of bias is observed while the false discovery rate is low in this analysis. Additionally, when patients are assessed on a regular frequency, but both false positives and false negatives are present (Scenario 4), these errors contribute large, yet *opposing*, amounts of bias that can cancel out. Under differing rates of sensitivity and specificity, it is even plausible that these errors could cancel out completely. This further raises an important cautionary point, that even if findings appear similar or unbiased between trial and RWD cohorts, biases due to measurement error may still be present at the individual level, and it is important to quantify them to contextualize such findings. However, while simulations suggest each type of bias may not substantially hinder comparability alone, it is unlikely that these phenomena exist in isolation in the real-world. When misclassified progression events and irregular assessment times occur simultaneously (Scenario 5), we have demonstrated that they can generate bias that is greater than the sum of their parts.

Note that these measurement differences may have varied effects in other disease settings with different event rates and may also depend on sample size and prognostic factors. While we assume in this illustrative simulation that the biases due to measurement error are not differential with respect to any baseline covariates, it is important for future work to study the identification and impact of important prognostic characteristics of measurement error. Furthermore, recall that we have defined these biases in RWD mPFS *in relation to* the “true” mPFS that would be observed in the control arm of a randomized trial. Future work should also examine how these biases impact treatment effect estimation (i.e., hazard ratio comparing PFS in a single-arm trial to PFS in an RWD comparator) under various effect sizes and trial outcomes.

In application, it can be challenging to quantify the amount of bias in PFS due to measurement error and definitively assess how much bias is attributed to each potential cause. In the context of MM, where real-world endpoints may be derived according to flexible algorithms using a subset of IMWG biomarkers typically observed in RWD, little is known about how such flexible algorithms perform relative to trial standards. Furthermore, different algorithms may have different false positive and negative rates, thereby yielding different amounts of measurement error and bias when compared to a trial. In this illustrative parametric simulation, we assume the performance of such flexible algorithms is already known (or estimated). Future studies should consider simulation designs that enable more direct performance evaluation of these algorithms relative to trial endpoints. Such studies would play an important role in understanding how measurement error manifests not just in theory, but in practice.

This paper highlights through simulations how measurement error and related biases may manifest and impact PFS, a time-to-event composite endpoint used in MM and oncology. Measurement error may present differently with other types of endpoints. For example, with endpoints based on binary outcomes, like overall response rate, surveillance bias may be less concerning, as it matters more *if* the outcome occurred rather than when in the study it occurred. Approaches to correct for outcome measurement error may differ for binary, continuous, or time-to-event outcomes and warrant further research (Carroll et al., 2006; Edwards et al., 2013; Kapetanakis et al., 2019; Innes et al., 2021; Zhu and Tang, 2022).

Lastly, while we have illustrated sources of measurement error in a NDMM population, mismeasured outcomes in other contexts and diseases are also common. The biases we have highlighted in this work are present and relevant across a wide range of therapeutic areas, both within and beyond oncology. Other relevant endpoints susceptible to measurement error bias may be, for example, based on imaging (Hong et al., 2012; Huang Bartlett et al., 2020; Ton et al., 2022; Mhatre et al., 2023) (i.e., in solid tumors) or patient-reported outcomes (Keogh et al., 2016). Simulation studies that quantify bias due to measurement error can be helpful tools when planning for ECA studies and can be used where possible for contextualizing study results in the presence of endpoint measurement differences. Improved understanding of the interplay between these biases in other diseases contexts may inform future approaches for mitigating measurement error biases and constructing more robust ECAs.

## Data Availability

Simulation code to generate data and conduct simulation study may be made available upon request. Inquiries can be directed to the corresponding author.
